# Editorial: Dietary Carbohydrate Digestibility and Metabolic Effects in Human Health

**DOI:** 10.3389/fnut.2019.00164

**Published:** 2019-10-15

**Authors:** Robert A. Rastall, F. Javier Moreno, Oswaldo Hernandez-Hernandez

**Affiliations:** ^1^Department of Food and Nutritional Sciences, The University of Reading, Reading, United Kingdom; ^2^Instituto de Investigación en Ciencias de la Alimentación, CIAL (CSIC-UAM), Universidad Autónoma de Madrid, Madrid, Spain

**Keywords:** prebiotic, dietary fiber, gut microbiota, bovine milk oligosaccharides, human milk oligosaccharides, enzymatic glycosylation, non-digestible carbohydrates, cross feeding

The links between some carbohydrate components of the human diet and health have been understood for decades. Beyond the provision of energy, digestible carbohydrates can have other impacts on host health including acting as dietary fiber and prebiotics. In order to qualify as either of these, carbohydrates need to be largely indigestible by the human gut (1). As discussed by Hernandez-Hernandez et al., current ways to measure the digestibility of carbohydrates *in vitro* remain challenging and are very poorly developed in relation to our ability to determine their impact on health in human trials. The most commonly used “*in vitro* digestion” methods (based on the use of microbial amylolytic enzymes combined with invertases) do not model the complex degradation of carbohydrate structures in the gut with any accuracy and these authors point the way toward using mammalian brush border enzyme extracts or, in the future, cloned mammalian enzymes (2).

One challenge in research of non-digestible carbohydrates and gut health is a lack of understanding of the functional ecology of the gut. At present, developments of novel non-digestible carbohydrates take place with a very limited knowledge of how they will be handled by the gut microbiome. Degradation and metabolism of dietary carbohydrates involve a complex multispecies action followed by metabolic cross feeding on the products. Currently, little is understood of the cross feeding networks that occur in the gut. In their review, Crost et al. reported on some of the details of cross feeding relationships involving starch degrading and mucin degrading *Ruminococcus* species. As degradation of carbohydrates involves bacterial glycoside hydrolases, further investigation of this interrelationship is needed. Study of the glycosyl hydrolases, expressed by specific gut bacteria under nutritional regimes is very challenging and will require the development of new techniques in the future. Blanco et al. discuss the application of *in silico* techniques to characterize the glycosidic capacity of a potentially beneficial bacterium, *Faecalibacterium prausnitzii*, potentially facilitating the design and manufacture of prebiotics targeted at specific organisms.

There are several prebiotic oligosaccharides on the market and the market leaders are the fructo-oligosaccharides and galacto-oligosaccharides, manufactured using enzymatic approaches. A review by Martins et al. reported the potential promise for the development of novel bioactive structures, and future alternate sources of nutritional carbohydrates that could be developed in future. This theme is expanded upon by Garcia-Valle et al. who evaluated the use of unripe plantain flour as a functional ingredient. Enzymatic glycosylation provides the potential to bring about synthesis of many novel bioactive molecules, such as novel prebiotic carbohydrates and derivatives of other nutritional compounds with improved functionality. An example of this is given by Gonzalez-Alfonso et al. who have used the enzyme cyclodextrin glucosyl transferase to α-glucosylate (-)-epigalocatechin gallate, to improve pH-, thermal stability, and antioxidant activity. Glycosylation of plant polyphenols may improve their activity and bioavailability more generically (3).

It is well-known that human milk contains an extremely complex array of oligosaccharide structures and these are believed to play an important role in infant nutrition, acting as prebiotic carbohydrates, anti-adhesive agents inhibiting the adhesion of pathogens and also acting as cell signaling molecules (4). It is, however, very difficult to carry out any kind of mechanistic study in infants and animal models are needed. Rudloff et al. describe the use of a pig model of necrotizing enterocolitis to study the metabolism of human milk oligosaccharides (HMO). They have used mixtures of oligosaccharides to study metabolism in the colon and excretion in urine. Demonstration that HMO can be absorbed, at least to some degree, from the human gut helps to explain some of the biological activities ascribed to HMO.

Despite the health benefits of HMO, it is not a trivial task to manufacture these oligosaccharides on a commercial scale. Only two human milk oligosaccharides have currently been put into large scale commercial production (i.e., 2′-fucosyllactose and lacto-N-neotetraose), although a number of new HMO products are in the commercial pipeline (5). Other non-digestible carbohydrates, however can mimic some of the effects of HMO (Verkhnyatskaya et al.) and galacto-oligosaccharides and fructo-oligosaccharides are both now added to infant formula. Verkhnyatskaya et al. explore the potential of other non-digestible carbohydrates to provide nutritional functionality in infant formulae in the future.

An alternative approach to the isolation or synthesis of human milk oligosaccharides might be to source complex oligosaccharides from bovine milk. As discussed by Robinson, these oligosaccharides are present in lower quantities than in human milk and the bovine oligosaccharides do not have exactly the same structures. They may, however, be more amenable to large scale extraction and they are being studied for their bioactivities and nutritional potential. For instance, Kuntz et al. describe the effect of bovine milk oligosaccharides from different cattle breeds on the growth of mammalian cells *in vitro*. The oligosaccharides had a dose-dependent growth inhibition effect on HT-29, Caco-2 and non-transformed human intestinal cells. They also induced differentiation in the non-transformed intestinal cells.

It is clear from the papers in this Research Topic that the field of non-digestible carbohydrates has many avenues to explore. The impact of such carbohydrate molecules on the gut microbiome is currently an area of intense investigation and hopefully will lead a detailed understanding of how specific carbohydrate structures can influence the ecology of the gut to produce specific metabolic signatures and how these can then impact on human health and wellbeing. It is becoming clear that dietary carbohydrates can also, at least *in vitro*, impact upon mammalian cell physiology. At the present time the extent and nature of metabolic cross feeding in the gut, the impact of microbial metabolites on human health and the degree to which direct interactions with cellular physiology occur *in vivo* in healthy humans are far from clear. However, pioneering *in vitro* approaches such as the culture of human gut microbiota in an anaerobic intestine-on-a-chip may serve as a cutting edge tool for mimicking the host-microorganism interactions in the human intestine (6, 7).

## Author Contributions

All authors listed have made a substantial, direct and intellectual contribution to the work, and approved it for publication.

### Conflict of Interest

The authors declare that the research was conducted in the absence of any commercial or financial relationships that could be construed as a potential conflict of interest.

## References

[1] Holscher HD. Dietary fiber and prebiotics and the gastrointestinal microbiota. Gut Microbes. (2017) 8:172–84. 10.1080/19490976.2017.1290756 28165863 PMC5390821

[2] Lee B-H Rose DR Lin AH-M Quezada-Calvillo R Nichols BL Hamaker BR. Contribution of the individual small intestinal α-glucosidases to digestion of unusual α-linked glycemic disaccharides. J Agric Food Chem. (2016) 64:6487–94. 10.1021/acs.jafc.6b01816 27480812

[3] Ilmberger N Rabausch U. Screening Glycosyltransferases for Polyphenol Modifications. In: Streit W Daniel R editors. Metagenomics. Methods in Molecular Biology, Vol 1539. New York, NY: Humana Press (2017). p. 229–36. 10.1007/978-1-4939-6691-2_14 27900693

[4] Akkerman R Faas MM de Vos P. Non-digestible carbohydrates in infant formula as substitution for human milk oligosaccharide functions: effects on microbiota and gut maturation. Crit Rev Food Sci Nutr. (2019) 59:1486–97. 10.1080/10408398.2017.1414030 29333864

[5] Bych K Miks MH Johanson T Hederos MJ Vigsnaes LK Becker P. Production of HMOs using microbial hosts - from cell engineering to large scale production. Curr Opin Biotech. (2019) 56:130–7. 10.1016/j.copbio.2018.11.003 30502637

[6] Kim HJ Li H Collins JJ Ingber DE. Contributions of microbiome and mechanical deformation to intestinal bacterial overgrowth and inflammation in a human gut-on-a-chip. Proc Natl Acad Sci USA. (2016) 113:E7–15. 10.1073/pnas.1522193112 26668389 PMC4711860

[7] Jalili-Firoozinezhad S Gazzaniga FS Calamari EL Camacho DM Fadel CW Bein A A complex human gut microbiome cultured in an anaerobic intestine-on-a-chip. Nat Biomed Eng. (2019) 3:520–31. 10.1038/s41551-019-0397-0 31086325 PMC6658209

